# A Global Comparison of Direct and Legacy Effects of Drought on Ecosystem Productivity

**DOI:** 10.1111/ele.70390

**Published:** 2026-05-03

**Authors:** Meng Liu, Steven A. Kannenberg, Josep Peñuelas, William R. L. Anderegg

**Affiliations:** ^1^ School of Biological Sciences University of Utah Salt Lake City Utah USA; ^2^ Wilkes Center for Climate Science and Policy University of Utah Salt Lake City Utah USA; ^3^ Department of Biology West Virginia University Morgantown West Virginia USA; ^4^ CREAF, Cerdanyola del Vallès Barcelona Catalonia Spain; ^5^ CSIC, Global Ecology Unit CREAF‐CSIC‐UAB, Bellaterra Barcelona Catalonia Spain

**Keywords:** carbon uptake, climate change, disturbance, drought legacy, drylands

## Abstract

Terrestrial ecosystems serve as a major carbon (C) sink, but the increasing frequency and intensity of drought threaten the C sink and ecological communities. A comprehensive understanding of the change in ecosystem productivity due to the direct effect versus the legacy effect of drought is still lacking. We quantify the magnitude change in terrestrial gross primary production (GPP) globally due to both direct and long‐term legacy effects. We find that the direct effect causes significant GPP decreases in magnitude of −5.94% [−13.40%, −3.21%] per year, while the legacy effect‐induced GPP change is weaker and non‐significant at a global scale. Drought legacy effects, however, are detectable in dry sub‐humid regions. The direct effect‐induced change is highly correlated with that of the legacy effect, and rooting depth is a key driver for both. These findings demonstrate the current resilience of global ecosystems to drought but underscore the long‐term vulnerability of dryland ecosystems.

## Graphical abstract

We quantify the magnitude change in terrestrial gross primary production (GPP) globally in response to direct and long‐term (4 years) legacy effects of drought. We find that the direct effect causes significant GPP decreases in magnitude of −6% per year, while the legacy effect‐induced GPP change is overall weaker and non‐significant in the long run.

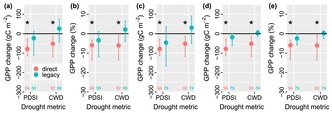

## 1 Introduction

Terrestrial ecosystems sequester approximately 2.0 Pg carbon (C) per year (net C sink) (Friedlingstein et al. 2023), counterbalancing ~20% of fossil fuel emissions. However, climate‐sensitive disturbances such as drought threaten terrestrial C sequestration (Anderegg, Wu, et al. 2022) by decreasing ecosystem productivity (Ciais et al. 2005; Dannenberg et al. 2022), increasing C losses from tree mortality (Anderegg, Hicke, et al. 2015), and altering ecosystem functioning and C cycling (Kannenberg et al. 2020). More frequent and severe droughts are projected in the following decades (Anderegg, Chegwidden, et al. 2022; Dai 2013) in response to climate change, potentially lessening the benefit of the terrestrial C sinks for climate change mitigation. Terrestrial gross primary production (GPP) is a critical metric of vegetation C assimilation, which is a major determinant of terrestrial C sink capacity and is drastically impacted by drought disturbances (Dannenberg et al. 2022). Hence, unravelling the changes and dynamics of terrestrial GPP and understanding how ecosystem C uptake responds to drought are paramount for terrestrial C sequestration, global C cycling, and climate change mitigation.

Drought exerts multiple effects on ecosystem productivity. GPP may decrease simultaneously during drought periods, a phenomenon often referred to as the direct effect of drought (Liu et al. 2024). Drought also has legacy effects, defined as alterations in ecosystem states or processes that persist after the drought has ceased (Müller and Bahn 2022). Under this broad definition, any change occurring after drought falls into the category of legacy effects. These effects might be caused by physiological damage (e.g., embolism) and structural or compositional changes (e.g., vegetation mortality). Previous research based on tree‐ring data indicated that tree growth might be suppressed for up to about 2–4 years after drought (Anderegg, Schwalm, et al. 2015). A recent study found that the sensitivity of GPP to water stress increased notably in dry regions following drought (Liu et al. 2025). Drought may cause strong reductions in GPP for as long as 2 years following drought, as documented by flux tower measurements in Germany (Pohl et al. 2023; Yu et al. 2022). All these observations are considered drought legacy effects under the broad definition outlined above. In this research, we focus on changes in the magnitude of GPP. Müller and Bahn (2022) reviewed and summarized the current understanding (until 2022) of drought legacy effects across species, community, and ecosystem levels and concluded that the drought legacy effects on GPP may last for a few months after drought. Both direct and legacy effects of drought are important sections of ecosystem carbon dynamics, and it is crucial to figure out the change in the magnitude of GPP due to both effects for long‐term C management, biodiversity conservation, and climate change mitigation.

However, previous research typically focused on either the direct effect or the legacy effect on GPP separately. For example, the direct effect caused GPP to decline by approximately 30% in the 2003 extreme drought across Europe (Ciais et al. 2005) and by more than 25% during the 2020 drought in the southwestern United States (Dannenberg et al. 2022), but the legacy effect was unresolved. The strong decrease in GPP in the northern midlatitude ecosystems due to direct drought effects was also detected, yet the legacy effect was not assessed (Gampe et al. 2021). A global analysis investigated the direct effect by combining flux tower data and remote sensing and found that remote sensing data tended to underestimate the magnitude of GPP change during drought periods (Stocker et al. 2019), yet legacy effects were not quantified. Other studies have examined the legacy effect on GPP solely (Müller and Bahn 2022), such as the change in the magnitude of GPP across North America following long‐term meteorological drought (Kannenberg et al. 2020). A recent study examined global postdrought GPP loss after drought, particularly flash drought, at an 8‐day scale and demonstrated that GPP could still decrease in an average of 32 days after drought, but the direct effect was not fully illustrated, nor were long‐term (longer than 1 year) responses assessed (Zhao et al. 2025). A case study investigated both direct and legacy effects on short‐term (i.e., seasonal) ecosystem productivity in response to the 2018 drought across Europe only (Bastos et al. 2020). A newly published research (Yu et al. 2025) quantified both direct and short‐term legacy effects across 76 flux tower sites in North America, Europe, and Australia. While this regional study offered valuable insights, a global understanding of these drought effects across multiple timescales and their correlations and mechanistic drivers needs further comprehensive investigation. A comprehensive, global comparison of how the direct and legacy effects affect GPP remains a major knowledge gap, and the drivers behind these effects are still poorly understood. Quantifying the difference between GPP changes driven by the direct versus the long‐term legacy effect and understanding their underlying mechanisms are crucial for modeling the impacts of drought disturbances on C cycling and for predicting future terrestrial C sinks in the twenty‐first century.

We analyzed the magnitude change in GPP during and after drought at a global scale and investigated the drivers of variability in the direct versus the long‐term legacy effects. We hypothesized that GPP could be lower than expected after drought due to the legacy effect. For simplicity, we referred to the magnitude change in GPP during and after drought as direct effect‐induced GPP change (ΔGPP_dir_) and legacy effect‐induced GPP change (ΔGPP_lag_), respectively. We used flux tower‐based GPP data at a global scale to represent ecosystem productivity (see Methods) and used two widely used drought metrics—the Palmer drought severity index (PDSI) (Palmer 1965) and climatic water deficit (CWD) (Abatzoglou et al. 2018) – to identify long‐term drought. This study focused on high severity drought events since low severity drought generally had limited impacts on ecosystems (Anderegg, Schwalm, et al. 2015; Liu et al. 2024). We defined ΔGPP_dir_ as the difference between GPP during drought and predrought GPP, and ΔGPP_lag_ was the difference between observed and expected GPP postdrought. We employed two methods to estimate the expected GPP, that is, using predrought GPP as the expected GPP and using random forest regression to predict the expected GPP. We compared ΔGPP_dir_ and ΔGPP_lag_ at a global scale and across land covers and investigated their drivers with generalized additive models. We sought to answer the following questions: (1) What is the difference between ΔGPP_dir_ and ΔGPP_lag_ at a global scale? (2) What is the difference and correlation between ΔGPP_dir_ and ΔGPP_lag_ across aridity levels and land cover types? (3) What are the dominant drivers of ΔGPP_dir_ and ΔGPP_lag_?

## 2 Materials and Methods

### 2.1 Data

**FIGURE 1:**
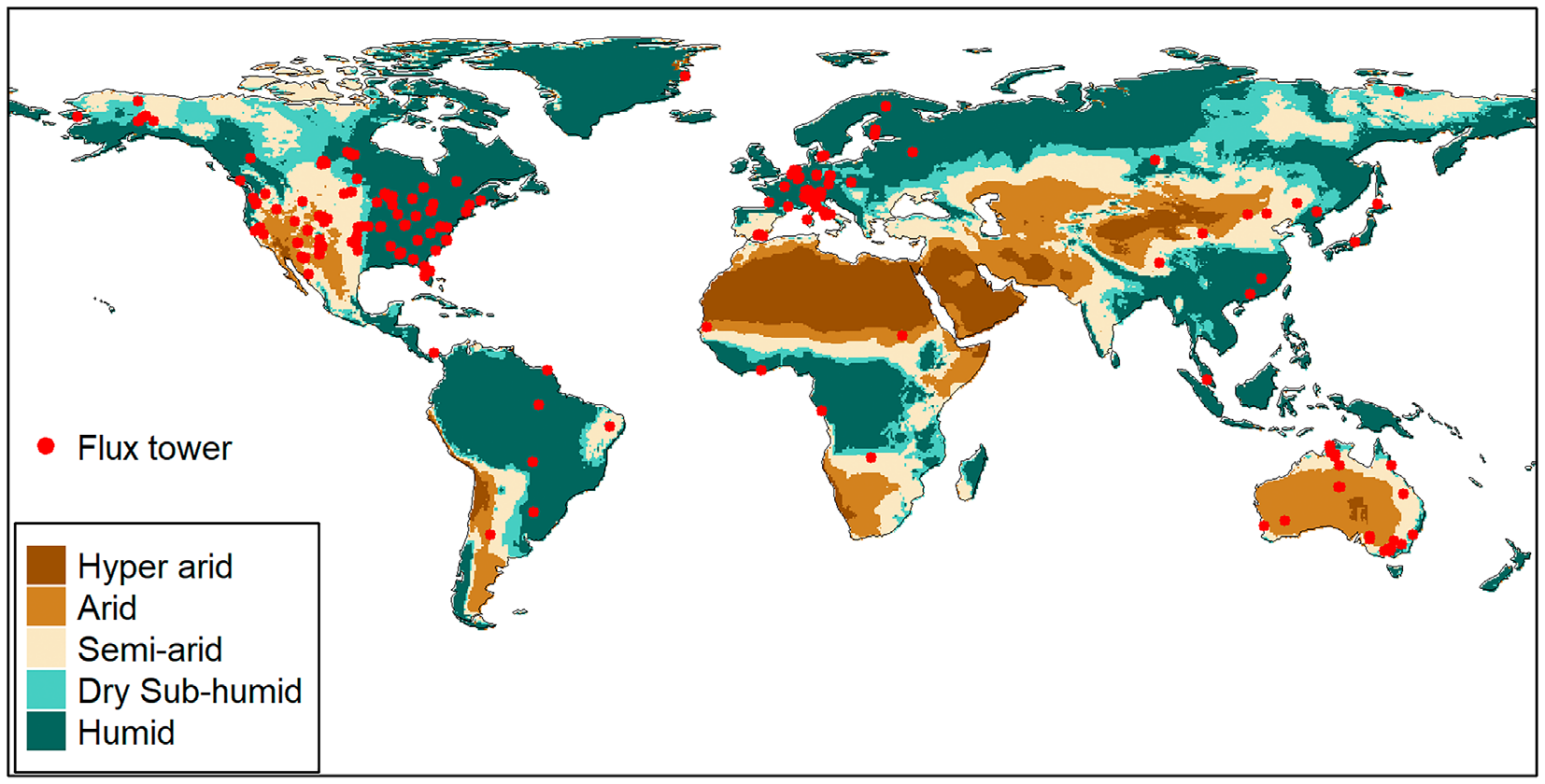
Distribution of the 247 flux tower sites. Each red dot indicates a flux tower. The aridity levels are defined based on the aridity index (AI): Hyper arid (AI < 0.05), arid (AI < 0.2), semi‐arid (AI < 0.5), dry sub‐humid (AI < 0.65), and humid (AI ≥ 0.65).

We used flux tower‐based gross primary production (GPP) data from both FLUXNET2015 (Pastorello et al. 2020) and AmeriFlux (https://ameriflux.lbl.gov/) to represent ecosystem productivity. FLUXNET2015 was produced with the ONEFlux processing pipeline and provided site‐level GPP measurements from 1991 to 2014 (the range varies based on sites) across the globe. AmeriFlux (Novick et al. 2018) provided a standardized dataset named ‘AmeriFlux FLUXNET’ following the same procedure as in FLUXNET2015. This dataset covered America only but had a longer duration, from 1991 to 2021 (at the time this study was initiated). For each flux tower site, we removed site‐years impacted by other disturbances, such as storms, wildfires, hurricanes, and herbicide treatments, based on the site descriptions. For example, the site US‐Vcm experienced a wildfire in 2013, so we only used site‐years before 2013 for this site. Based on the International Geosphere–Biosphere Programme (IGBP) classification of each site, we removed sites in croplands and non‐vegetation (permanent wetlands, water, snow, urban, and barren). After sieving these data, we finally obtained 247 sites (Figure 1; Table S1) and 1913 site‐years for the following analyses. The median (average) duration of these available flux tower data was 5 (7.7) years. It is understandable that there are still substantial gaps in the global sampling of the flux tower sites due to budget and accessibility limits, and more long‐term study sites are needed in South America, Africa, and Asia to generate truly global coverage.

We leveraged two widely used drought indices from literature, that is, the Palmer Drought Severity Index (PDSI) (Palmer 1965) and climatic water deficit (CWD) (Abatzoglou et al. 2018), to identify drought events, to be specific, meteorological drought. We extracted site‐level PDSI and CWD using the coordinates of each flux tower site. PDSI is a standardized metric derived from a two‐layer soil water balance model, where negative values indicate dry conditions and positive values indicate wet. We downloaded monthly 4‐km historical PDSI data from TerraClimate (Abatzoglou et al. 2018), which were based on the Penman–Monteith equation‐derived potential evapotranspiration (PET). Monthly PDSI data were averaged to generate full‐calendar‐year annual PDSI data. Drought disturbances were defined as annual PDSI values below −3 (Liu et al. 2025; Palmer 1965), which indicates long‐term severe drought. CWD (the difference between PET and ET) was also downloaded from TerraClimate, where high CWD values indicate dry conditions. Monthly 4‐km CWD was aggregated to the annual scale by taking the sum of the 12 months. We used the site‐level 95th quantile (q_95_) of CWD from the past four decades as a threshold for each site to identify drought, i.e., CWD > q_95_ indicates a long‐term drought event.

### 2.2 Quantification of the Change in GPP

We defined the magnitude change in GPP during and after drought as direct effect‐induced change (ΔGPP_dir_) and legacy effect‐induced change (ΔGPP_lag_), respectively. We calculated the two types of change using Equation (1), in which GPP_drought_, GPP_predrought_, and GPP_postdrought_ were the mean annual GPP during drought, predrought, and postdrought years (Table S2), respectively. GPP_expected_ was the GPP we expected, assuming the drought did not occur. We used the three‐year average drought‐free GPP (Schwalm et al. 2017; Yan et al. 2025) between the last and the current drought as GPP_predrought_ (Figure S1) to constrain interannual variability. The average GPP could also be used when there were less than 3 years. For example, when there were only two drought‐free years before the current drought, we would use a two‐year average GPP. GPP_predrought_ would be NA if no drought‐free data were available. GPP_drought_ was the mean annual GPP during drought years. Continuous drought years (e.g., a two‐year drought) were regarded as one drought event. GPP_postdrought_ was the four‐year average drought‐free GPP after the current drought and before the next drought. We used 4 years here because the long‐term drought legacy effect can last for as long as 4 years based on previous research (Anderegg, Schwalm, et al. 2015; Liu et al. 2025). We would still use GPP_postdrought_ when there were less than 4 years available (e.g., only 2 years), and GPP_postdrought_ would be NA if no data were available at all.

(1) $$\left\{\begin{array}{l} \Delta {\mathrm{GPP}}_{\mathrm{dir}}={\mathrm{GPP}}_{\text{drought}}-{\mathrm{GPP}}_{\text{predrought}}\\ \Delta {\mathrm{GPP}}_{\mathrm{lag}}={\mathrm{GPP}}_{\text{postdrought}}-{\mathrm{GPP}}_{\text{expected}} \end{array}\right.$$

We used two methods to estimate GPP_expected_. In the first method, we used GPP_predrought_ to represent GPP_expected_ (i.e., GPP_expected_ = GPP_predrought_). This method assumed that GPP_predrought_ was reflective of postdrought GPP (Schwalm et al. 2017). In the second method, we used random forest regression to predict GPP_expected_. Random forest regression exerts no statistical assumptions on data and is well‐suited for analyzing high‐dimensional data with complex structure. We used annual flux tower GPP data to train the random forest model using the “*randomForest*” package in R. We selected flux tower GPP before the first drought as the response variable so that the samples were not impacted by drought. In this way, random forest prediction could represent the GPP data free of drought disturbance. The predictors contained many climatic, nutrient, and biological variables, including PDSI, downward surface shortwave radiation (Srad), vapor pressure deficit (VPD), temperature (T), precipitation (P), soil moisture (SM), leaf area index (LAI) (Cao et al. 2023), IGBP land cover, soil organic carbon (SOC), soil nitrogen (Soil.N), and soil cation exchange capacity (Soil.CEC). Climatic variables such as 4‐km Srad, T, P, and PDSI were downloaded from TerraClimate and aggregated to the annual temporal resolution. The 0.1° soil moisture data (0–100 cm) from the European Center for Medium‐Range Weather Forecasts Reanalysis v5 (ERA5) were downloaded and resampled to 4 km (nearest neighbor). The depth of soil moisture used (integrated over 0–100 cm) was the same for all sites. The 8‐km Global Inventory Modelling and Mapping Studies (GIMMS) LAI4g data were resampled to 4 km (nearest neighbor). The 250‐m SOC, Soil.N, and Soil.CEC were obtained from SoilGrids250m (https://soilgrids.org/) and aggregated to 4 km. We extracted site‐level predictors using the coordinates of all flux tower sites and used data from all available sites to train the random forest model, producing a universal model for our global analysis. We used 500 decision trees for computational efficiency, and the number of split (i.e., mtry in ‘*randomForest*’) was determined based on the accuracy of the model. To be specific, we firstly separated all predrought samples into a training dataset (70% of samples) and a testing dataset (the rest 30%) randomly. With the training dataset, we tried all possible values for mtry and chose the one with the best accuracy. The best mtry turned out to be 3, with *R* ^2^ = 0.75, slope = 1.06, and bias = 1.92 gC m^−2^ per year. With the testing dataset, we checked the accuracy of the trained model, *R* ^2^ = 0.72, slope = 1.03, and bias = 6.95 gC m^−2^ per year (Figure S2). With the well‐trained random forest model, we used out‐of‐bag predictions for both the training and testing data for the following analyses. The random forest model was trained using the predrought samples (training set + testing set) only, and for postdrought GPP, we used the well‐trained model to predict GPP with postdrought LAI, Srad, T, P, SM, and other variables mentioned above.

For flux tower site‐level data, we used a bootstrapping strategy to check the significance of the change in GPP because the sample size was very small. We used the “*boot*” package in R to produce 1000 simulations and calculated the confidence intervals of ΔGPP_dir_ and ΔGPP_lag_, respectively. When the confidence intervals crossed zero, the change in GPP was not significant; otherwise, we used an asterisk (*) to indicate a significant change. We quantified ΔGPP_dir_ and ΔGPP_lag_ at a global scale and across aridity levels as well as land cover types. The global 1‐km aridity index (AI) version 3 (Zomer et al. 2022) was downloaded from figshare (https://figshare.com). There were five aridity levels used: hyper arid (AI < 0.05), arid (AI < 0.2), semi‐arid (AI < 0.5), dry sub‐humid (AI < 0.65), and humid regions (AI ≥ 0.65).

### 2.3 Drivers of the Change in GPP

We used the generalized additive model (GAM) implemented in the “*mgcv*” package in R to investigate the drivers of the change in GPP. GAM is a widely used statistical model, which can capture the potential nonlinear correlations between variables and identify those significant drivers. We used separate GAMs for ΔGPP_dir_ and ΔGPP_lag_, respectively, because their drivers were different. For legacy effect‐induced change, the response variable was ΔGPP_lag_, and the predictors were various climatic and ecophysiological variables, including postdrought PDSI (PDSI_postdrought_), postdrought Srad (Srad_postdrought_), postdrought VPD (VPD_postdrought_), postdrought T (T_postdrought_), postdrought P (P_postdrought_), postdrought SM (SM_postdrought_), postdrought LAI (LAI_postdrought_), ΔPDSI_post‐pre_ which was postdrought PDSI minus predrought PDSI, ΔSrad_post‐pre_, ΔVPD_post‐pre_, ΔT_post‐pre_, ΔP_post‐pre_, ΔSM_post‐pre_, ΔLAI_post‐pre_, SOC, Soil.N, Soil.CEC, Aridity Index (AI), species diversity (diversity) (Ellis et al. 2012), specific leaf area (SLA) (Moreno‐Martínez et al. 2018), rooting depth (root.depth) (Stocker et al. 2023), root shoot ratio (root.shoot) (Ma et al. 2021), wood density (wood.density) (Yang et al. 2024), forest canopy height (canopy.height) (Simard et al. 2011), and IGBP land cover (land.cover). Postdrought PDSI was the four‐year average PDSI after the current drought and before the next drought, and predrought PDSI was the three‐year average PDSI between the last and the current drought. (Refer to definitions of GPP_postdrought_ and GPP_predrought_ in the previous section.) The same definition worked for other predictors. For direct effect‐induced change, the response variable was ΔGPP_dir_, and the predictors were similar with only a few changes, such as using mean annual PDSI during drought (PDSI_drought_) rather than PDSI_postdrought_ as a predictor and using ΔPDSI_drought‐pre_ (PDSI_drought_ minus predrought PDSI) rather than ΔPDSI_post‐pre_. The same change worked for Srad, VPD, T, P, SM, and LAI. We used flux tower‐based annual GPP data and extracted the predictors using the coordinates of these sites. Both response variables and predictors were standardized to remove their units (i.e., using *z*‐score). We employed a stepwise routine to remove non‐significant predictors and train the GAMs by removing the least important predictor one at a time. We checked the concurvity of the final model and made sure the value was lower than 0.8 as a rule of thumb. The importance of the finally maintained predictors was the percentage of the individual effect of each predictor towards the explained variance derived from the GAM models.

## 3 Results

### 3.1 Global Direct and Legacy Effects of Drought on the Magnitude of GPP

**FIGURE 2:**
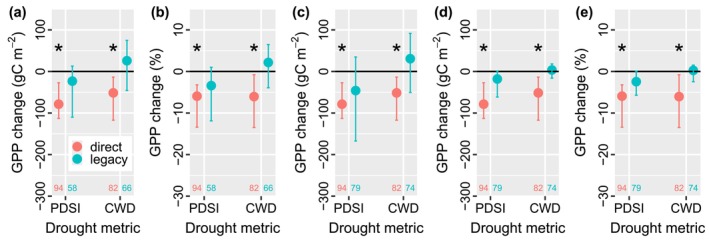
Weak legacy effects of drought on the magnitude of GPP. (a, b) The direct effect‐induced (ΔGPP_dir_) and legacy effect‐induced (ΔGPP_lag_) (a) change in GPP and (b) relative change in GPP when using predrought GPP as the expected GPP. (c–e) Use random forest (RF) regression to estimate the expected GPP and further calculate ΔGPP_dir_ and ΔGPP_lag_ based on (c) GPP, (d) GPP anomaly, and (e) relative change, respectively. Three years predrought and 4 years postdrought are used. The dots are the median changes, and the error bars are the corresponding 95% confidence intervals from bootstrapping. Asterisks (*) indicate the confidence intervals do not cross zero. The numbers at the bottom of each subfigure are the sample size.

The direct effect induced significant GPP decreases, while the long‐term legacy effect had no significant impacts on the magnitude of GPP at a global scale. When using predrought GPP as the expected GPP, ΔGPP_dir_ was significantly lower than zero for PDSI and CWD derived drought, at −78.65 (Confidence Interval = [−113.08, −27.18]) and −51.47 [−117.21, −13.42] gC m^−2^ per year, respectively (Figure 2a). ΔGPP_lag_ was not significant for drought derived from the two metrics, −23.18 [−109.96, 12.92] and 25.90 [−45.67, 74.88] gC m^−2^ per year, respectively. We calculated relative change by dividing the change in the magnitude of GPP by the mean annual GPP, where ΔGPP_dir_ was still significant for PDSI and CWD, −5.94% [−13.40%, −3.21%] and −6.04% [−13.49%, −0.78%], respectively, and ΔGPP_lag_ was not significant for both metrics, −3.40% [−11.88%, 1.0%] and 2.17% [−3.92%, 6.48%] (Figure 2b). When using random forest regression to predict the expected GPP (Figure 2c), ΔGPP_lag_ was not significant for both PDSI and CWD, and ΔGPP_dir_ was the same as that in Figure 2a because ΔGPP_dir_ relied on GPP during and pre drought only rather than the expected GPP. With the random forest‐predicted GPP, we further derived GPP anomalies (Figure 2d) and relative change (Figure 2e). We subtracted the mean annual GPP from the GPP data for each flux tower site to produce GPP anomalies and recalculated the change in GPP (Figure 2d), where ΔGPP_lag_ was not significant for both metrics. The ΔGPP_lag_ values derived from GPP anomalies (Figure 2d) and from predrought GPP (Figure 2a) were comparable and significantly correlated (*R* ^2^ = 0.42, *p* < 0.0001; Figure S3), and their correlation was higher than using ΔGPP_lag_ values in Figure 2a,c. Therefore, we used GPP anomalies for the following analyses. We calculated relative change with GPP anomalies (Figure 2e), where ΔGPP_dir_ was significantly negative for both metrics, and ΔGPP_lag_ was still non‐significant.

**FIGURE 3:**
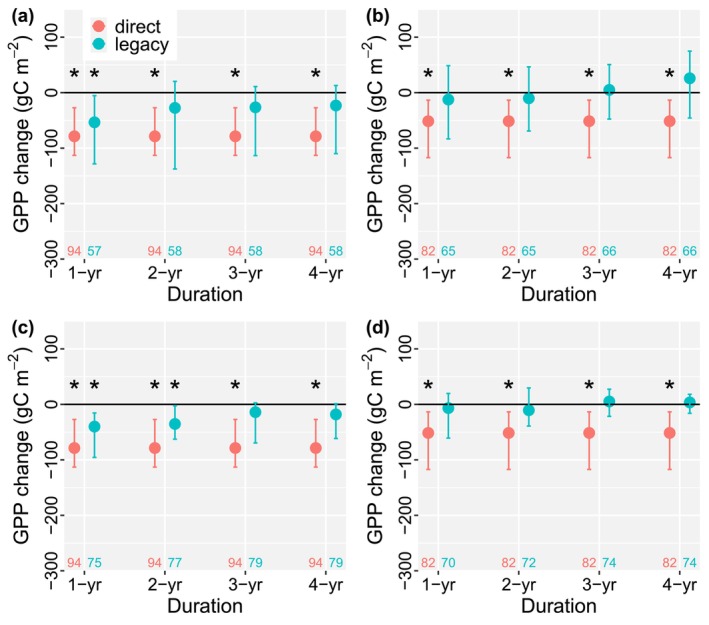
Comparison of 1, 2, 3, and 4‐year legacy effects on GPP. (a, b) Direct and legacy effects induced GPP change when using predrought GPP as the expected GPP and using (a) PDSI and (b) CWD to identify drought, respectively. Different durations, including 1, 2, 3, and 4‐year average change in postdrought GPP, are used for comparisons. (c, d) Change in GPP when using random forest regression to estimate the expected GPP and using (c) PDSI and (d) CWD to identify drought. The dots are the median changes, and the error bars are the corresponding 95% confidence intervals. Asterisks (*) indicate the confidence intervals do not cross zero. The numbers at the bottom are the sample size.

We also quantified the drought legacy effect globally using different durations of postdrought periods of 1, 2, 3, and 4 years. For droughts derived from both PDSI and CWD, ΔGPP_lag_ was mostly negative and not statistically significant (Figure 3). An increasing trend was observed as the postdrought period extended from 1 to 4 years; for example, ΔGPP_lag_ changed from −53.46 to −23.18 gC m^−2^ per year in Figure 3a, indicating a gradual recovery of GPP after drought. Notably, ΔGPP_lag_ showed significant decreases during the first year after drought events (short‐term legacy) based on PDSI data (Figure 3a,c). This is consistent with previous research suggesting that GPP may take 1 or 2 years to fully recover after drought (Schwalm et al. 2017). In contrast, ΔGPP_lag_ calculated from CWD‐derived droughts exhibited some negative changes, but these were not statistically significant, possibly because PDSI reflects the long‐term accumulation of drought stress, whereas CWD focuses on immediate water deficits and does not account for previous drought conditions. The results were similar when using relative change in GPP (Figure S4), with most cases showing non‐significant legacy effects, except the short‐term change, that is, the first 1 or 2 years following drought.

### 3.2 Drought Effects Vary Across Climates and Land Covers

**FIGURE 4:**
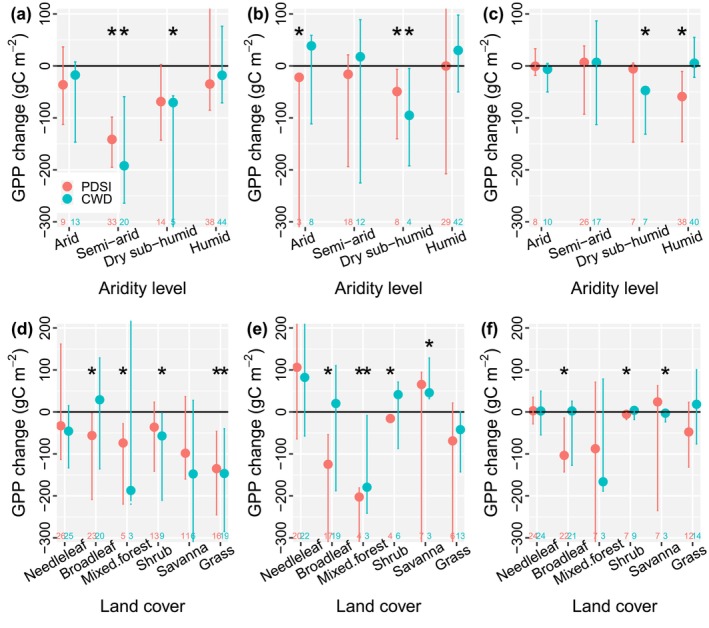
Both direct and legacy effects induced significant GPP decreases in dry regions. (a–c) Change in GPP across aridity levels due to (a) the direct effect, (b) legacy effect based on predrought GPP, and (c) legacy effect based on random forest predicted GPP. (d–f) Change in GPP across land cover types due to (d) the direct effect, (e) legacy effect based on predrought GPP, and (f) legacy effect based on random forest predicted GPP. Three years predrought and 4 years postdrought are used. The dots are the median changes, and the error bars are the 95% confidence intervals. Asterisks (*) indicate the confidence intervals do not cross zero. The numbers at the bottom are the sample size. Needleleaf forests contain evergreen and deciduous needleleaf forests in IGBP classification; broadleaf forests contain evergreen and deciduous broadleaf forests; shrublands contain closed and open shrublands; savannas contain savannas and woody savannas. Mixed forests and grasslands are the same as those in IGBP.

Both direct and legacy effects induced significant decreases in the magnitude of GPP in some dry regions. ΔGPP_dir_ was significantly lower than zero in semi‐arid regions via both PDSI and CWD and in dry sub‐humid regions for CWD, while the GPP decreases in other regions were not significant (Figure 4a). When using predrought GPP as the expected GPP, ΔGPP_lag_ was significantly lower than zero in arid and dry sub‐humid regions for PDSI and in dry sub‐humid regions for CWD (Figure 4b), and it was not significant in other cases. When using random forest predicted GPP as the expected GPP, ΔGPP_lag_ was significantly negative in dry sub‐humid regions for CWD and in humid regions for PDSI (Figure 4c). The legacy effect induced significant GPP decreases in dry sub‐humid regions for both methods, and the direct effect induced change was also significant in dry regions, such as semi‐arid and dry sub‐humid regions. Among land cover types, ΔGPP_dir_ was significantly negative in broadleaf forests, mixed forests, shrublands, and grasslands (Figure 4d). For ΔGPP_lag_, the GPP decrease was significant in broadleaf forests, mixed forests, and shrublands when using predrought GPP as the expected GPP (Figure 4e). GPP increased significantly in savannas when using CWD to identify drought, indicating postdrought recovery and enhanced productivity. When using random forest predicted GPP as the expected GPP, ΔGPP_lag_ was significantly negative in broadleaf forests, shrublands, and savannas (Figure 4f). The legacy effect‐induced GPP decrease was significant in broadleaf forests and shrublands when using the two methods. The results were quite similar when using relative change in GPP, where dry regions such as dry sub‐humid regions exhibited significant decreases in both ΔGPP_dir_ and ΔGPP_lag_ (Figure S5).

### 3.3 Drivers of the Direct and Legacy Effects

**FIGURE 5:**
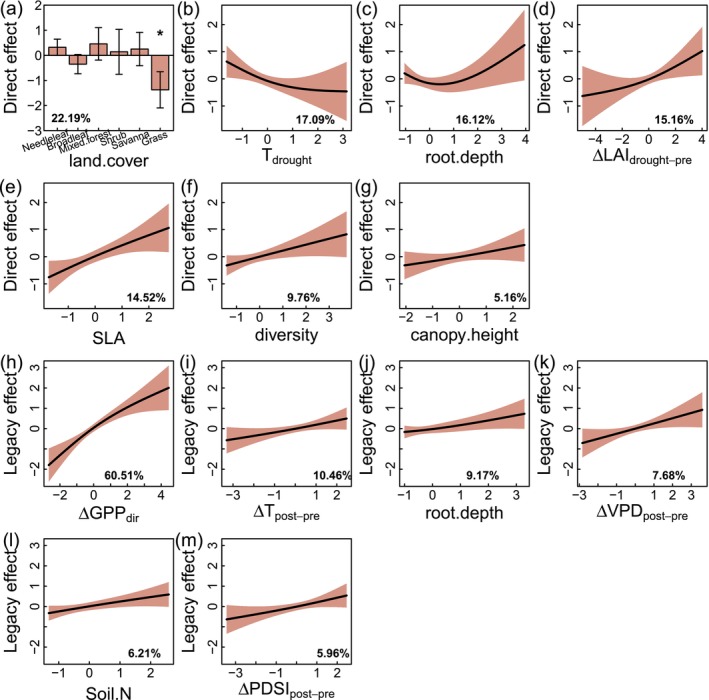
The direct effect is the major driver of the legacy effect‐induced change in GPP. (a–g) Seven significant drivers of the direct effect (ΔGPP_dir_): Land cover (land.cover), temperature during drought (T_drought_), root.depth, change in leaf area index (ΔLAI_drought‐pre_ = LAI during drought − predrought LAI), specific leaf area (SLA), species diversity (diversity), and forest canopy height (canopy.height). All variables are standardized (*z*‐score) to remove units. The percentages in the subfigures are the relative importance. Three years predrought and 4 years postdrought are used. The error bars in subfigure (a) are confidence intervals. (h–m) Six significant drivers of the legacy effect (ΔGPP_lag_): Direct effect‐induced change (ΔGPP_dir_), change in temperature (ΔT_post‐pre_ = postdrought T − predrought T), rooting depth (root.depth), change in vapour pressure deficit (ΔVPD_post‐pre_), soil nitrogen (Soil.N), and change in PDSI (ΔPDSI_post‐pre_).

For direct effect‐induced change, ΔGPP_dir_, land cover was the most important predictor. We used a generalized additive model (GAM) to investigate the drivers influencing the change in the magnitude of GPP. For the direct effect, the response variable was the average ΔGPP_dir_ from the two drought metrics and two methods mentioned above. Based on GAM, the fitted *R* ^2^ was 0.38, and there were seven significant drivers: land cover (land.cover), temperature during drought (T_drought_), rooting depth (root.depth), change in leaf area index (ΔLAI_drought‐pre_ = LAI during drought − predrought LAI), specific leaf area (SLA), species diversity (diversity), and forest canopy height (canopy.height) in a descending order of importance (Figure 5a–g; Table S3). Across land cover types, ΔGPP_dir_ exhibited significant decreases in grasslands (Figure 5a), and the changes in other types were not significant. High temperature during drought was related to the decrease in GPP (ΔGPP_dir_ < 0) (Figure 5b), indicating that heat stress compounded with water stress constrained photosynthesis. Rooting depth was generally positively correlated with ΔGPP_dir_ (Figure 5c), emphasizing that deep roots contributed to increasing drought resistance. ΔLAI_drought‐pre_ was positively correlated with ΔGPP_dir_ (Figure 5d), which means increased LAI was related to increased GPP (ΔGPP_dir_ > 0). High specific leaf area was related to the increase in GPP (Figure 5e). Species diversity was positively correlated with ΔGPP_dir_, and high diversity contributed to the increase in GPP during drought (Figure 5f), indicating that high diversity can enhance ecosystem resistance to drought (Isbell et al. 2015). GPP may increase during drought with increased canopy height (Figure 5g), which might be because higher canopy height is related to deeper roots to access more water (Chen et al. 2024).

For the legacy effect‐induced GPP change, ΔGPP_lag_, the direct effect‐induced change (i.e., ΔGPP_dir_) was the most important predictor, suggesting that these two drought effects were actually coupled. For the legacy effect, the fitted *R* ^2^ of the GAM was 0.49, and there were six significant drivers, including ΔGPP_dir_, change in temperature (ΔT_post‐pre_ = postdrought T − predrought T), rooting depth, change in vapour pressure deficit (ΔVPD_post‐pre_), soil nitrogen (Soil.N), and change in PDSI (ΔPDSI_post‐pre_) (Figure 5h–m; Table S3) in descending order of importance. There was a positive correlation between ΔGPP_dir_ and ΔGPP_lag_ (Figure 5h), indicating that a strong direct effect was related to a strong legacy effect. When there was a greater drop in GPP during drought periods, the legacy effect‐induced decrease in GPP also tended to be larger. ΔT_post‐pre_ exhibited a positive correlation with ΔGPP_lag_ (Figure 5i), suggesting that higher temperature postdrought might contribute to GPP recovery. Rooting depth was positively correlated with ΔGPP_lag_ (Figure 5j), that is, deeper roots promoted GPP recovery, which was consistent with the result in ΔGPP_dir_. Higher postdrought VPD also contributed to increased GPP (Figure 5k), which might be due to fewer clouds and more incoming solar radiation. High nitrogen content contributed to increased GPP after drought (Figure 5l), suggesting the benefits of high nutrients in ecosystem recovery. Increased PDSI (i.e., increased water availability) after drought was beneficial to the increase in GPP after drought (ΔGPP_lag_ > 0) (Figure 5m).

## 4 Discussion

Climate‐sensitive disturbances, such as drought, significantly impact terrestrial ecosystems, ecological communities, and land carbon uptake. We quantified and compared changes in the magnitude of GPP induced by both the direct and legacy effects of long‐term drought. The direct effect induced significant decreases in GPP at a global scale. In contrast, the legacy effects were generally weak; that is, the GPP change induced by long‐term legacy effects was mostly non‐significant. This finding aligns with previous regional research (Kannenberg et al. 2019, 2020), which also found marginal and non‐significant drought legacy effects on GPP in North America. This suggests that, on average, GPP can fully recover within the annual time frame after drought, restoring the ecosystem's capacity to assimilate carbon to pre‐drought levels in many regions. Varying the length of post‐drought periods (e.g., 1, 2, 3, and 4‐year) yielded similar results (Figure 3), indicating the robustness of our findings. Notably, we observed some short‐term legacy effects of drought, with significant GPP decreases (ΔGPPlag < 0) in the first year after drought (Figure 3a,c). This is consistent with the reported one‐year (or two‐year) recovery of ecosystem GPP after drought (Müller and Bahn 2022; Pohl et al. 2023; Schwalm et al. 2017). While drought has pervasive and long‐lasting legacy effects on tree‐ring growth (Anderegg, Schwalm, et al. 2015), it is noteworthy that the legacy effect of drought on long‐term ecosystem GPP is relatively weak on global average. Results using growing season productivity only are comparable (Figure S6), with non‐significant legacy effects globally. In summary, ecosystem GPP demonstrates high resilience to drought, with only weak long‐term legacy effects observed on the magnitude of post‐drought GPP.

We also find that ecosystems in dry regions tend to exhibit decreased GPP in response to the legacy effect. Among aridity levels, the change in the magnitude of GPP due to the legacy effect is significant in dry sub‐humid regions (Figure 4b,c), where ΔGPP_lag_ is significantly lower than zero when using both predrought GPP and random forest prediction to represent the expected GPP. This decrease in GPP due to the legacy effect in dry regions aligns with the GAM analysis showing that rooting depth and change in PDSI are key drivers (Figure 5j,m), both of which are extremely important in these water‐limited ecosystems. Deep roots and increased PDSI contribute to accessing more water and promoting ecosystem recovery after drought. In line with the fact that the direct effect‐induced change (ΔGPP_dir_) is significantly and positively correlated with ΔGPP_lag_ (Figure 5h), the decrease in GPP due to the direct effect is also significant in dry regions, such as semi‐arid and dry sub‐humid regions (Figure 4a). For the direct effect‐induced change in GPP (Figure 5a–g), temperature, rooting depth, change in LAI, and species diversity are all important drivers, where low temperature, deep roots, high diversity, and increased leaf area contribute to the increase in productivity during drought. Water sensitive regions such as grasslands exhibit strong and significant decreases in GPP in response to the direct effect. Our results demonstrate the detectable GPP decreases in some dry regions due to both direct and legacy effects of drought, highlighting the vulnerability of dryland ecosystems. With more drought being projected under future warming climates (Dai 2013), ecosystems in dry regions may become more vulnerable, threatening ecosystem sustainability and long‐term terrestrial C sinks.

Our research provides a novel advance via an integrated assessment of the long‐term impacts of both direct and legacy effects of drought on ecosystem productivity and the corresponding drivers at a global scale. Previous research either focused on the drought legacy effect (Kannenberg et al. 2020) or the direct effect (Dannenberg et al. 2022), and the drivers and correlations of the change in the magnitude of GPP due to these two effects were not discussed globally. This research fills a key gap and quantifies both direct and long‐term legacy effects of drought, highlighting that the direct effect could lead to an ~6% decrease in annual GPP on average, while the legacy effects exert non‐significant impacts at a global scale (Figure 2b). We further investigate the drivers of the drought effects and find that rooting depth was an important factor affecting both effects. This comprehensive approach is crucial for a holistic understanding of ecosystem responses to drought. Second, our research demonstrates that the direct effect and the legacy effect are actually coupled, where the smaller the ΔGPP_dir_ (i.e., more damage during drought periods), the lower the ΔGPP_lag_ (Figure 5h). Large damage during drought periods drives strong legacy effects postdrought to some extent. We also find that dry regions, particularly dry sub‐humid regions, were vulnerable to both direct and legacy effects, where the legacy effects were still significant even 4 years after drought (Figure 4a–c). These results emphasize the importance of considering both effects for effective ecosystem management, biodiversity conservation, and C cycle modeling. Third, previous research (Müller and Bahn 2022; Zhao et al. 2025) tended to focus on short‐term (daily to monthly) drought legacy effects, which might be because short‐term effects are stronger than long‐term legacy effects, as explained in Figure 3a. However, our research highlights that drought can still exert long‐lasting impacts on ecosystem productivity, particularly in dry regions. This finding challenges the assumption that legacy effects are necessarily short‐lived and underscores the need for long‐term monitoring and modeling to fully capture the consequences of drought events. These innovative results harmonize both short‐term and long‐term drought effects, which is great progress moving forward in promoting our understanding of drought impacts on terrestrial ecosystems.

## Author Contributions

Meng Liu and William R.L. Anderegg conceptualized the study. Meng Liu designed and improved the study with input from all co‐authors. Meng Liu wrote the initial draft, and Steven A. Kannenberg, Josep Peñuelas, and William R.L. Anderegg discussed the design, analyses and results and provided valuable comments and revisions.

## Funding

William R. L. Anderegg acknowledges support from the David and Lucille Packard Foundation and US National Science Foundation grants 2003017, 2044937, 2330582, and Alan T. Waterman Award IOS‐2325700. Steven A. Kannenberg was supported by the US National Science Foundation Division of Environmental Biology award #2331162 and US National Science Foundation Dynamics of Integrated Socio‐Environmental systems award #2408954. Josep Peñuelas was supported by the PID2022‐140808NB‐I00 grant funded by MCIN of Spain and the European Union NextGeneration EU/PRTR and by the Catalan Government grant AGAUR2023 CLIMA 00118.

## Supporting information


**Figure S1:** A schematic of pre‐ and postdrought data. There are three predrought years and four postdrought years to derive the change in the magnitude of GPP due to the direct and legacy effects.
**Figure S2:** Accuracy of random forest regression for the training and testing datasets. Comparison of observed GPP from flux tower data and predicted GPP from random forest regression, where bias is the observed GPP minus the predicted GPP. *p* value comes from linear regression.
**Figure S3:** Comparison of the legacy effect‐induced change in GPP (ΔGPPlag) based on predrought GPP and random forest prediction. (a, b) ΔGPPlag based on predrought GPP versus ΔGPPlag based on (a) GPP anomaly and (b) GPP from random forest regression. GPP anomaly is calculated using annual GPP minus the average GPP for each site. The *p* values are based on linear regression.
**Figure S4:** Comparison of 1, 2, 3, and 4‐year legacy effect‐induced relative change in GPP. (a, b) Direct and legacy effects induced relative change when using predrought GPP as the expected GPP and using (a) PDSI and (b) CWD to identify drought. Different durations, including 1, 2, 3, and 4‐year, are used for comparisons. (c, d) Relative change in GPP when using random forest regression to estimate the expected GPP and using (c) PDSI and (d) CWD to identify drought. The dots are the median changes, and the error bars are the corresponding 95% confidence intervals. Asterisks (*) indicate the confidence intervals do not cross zero. The numbers at the bottom are the sample size.
**Figure S5:** Relative change in GPP in response to direct and legacy effects across land covers. (a–c) Relative change in GPP across aridity levels due to (a) the direct effect, (b) legacy effect based on predrought GPP, and (c) legacy effect based on random forest predicted GPP. (d–f) Relative change in GPP across land cover types due to (d) the direct effect, (e) legacy effect based on predrought GPP, and (f) legacy effect based on random forest predicted GPP. Three years predrought and 4 years postdrought are used. The dots are the median changes, and the error bars are the corresponding 95% confidence intervals. Asterisks (*) indicate the confidence intervals do not cross zero. The numbers at the bottom are the sample size.
**Figure S6:** Direct and legacy effects on the magnitude of growing season GPP. (a, b) The direct effect induced (ΔGPPdir) and legacy effect‐induced (ΔGPPlag) (a) change in GPP and (b) relative change in GPP when using predrought GPP as the expected GPP. (c, d) Use random forest (RF) regression to estimate the expected GPP and further calculate ΔGPPdir and ΔGPPlag based on (c) GPP anomaly and (d) relative change. Four years postdrought are used. The growing season is from April to October in the Northern Hemisphere (> 23.5° N), October to April in the Southern Hemisphere (< 23.5° S), and the whole year in the tropics. GPP, PDSI, and CWD are all extracted from the growing season. Three years predrought and 4 years postdrought are used. The dots are the median changes, and the error bars are the corresponding 95% confidence intervals. Asterisks (*) indicate the confidence intervals do not cross zero. The numbers at the bottom are the sample size.
**Table S1:** Flux tower sites from FLUXNET2015 and AmeriFlux FLUXNET.
**Table S2:** A summary of variables used in the manuscript.
**Table S3:** Results of the generalized additive models (GAM) for the direct and indirect effect‐induced GPP change.

## Data Availability

The climatic data are from TerraClimate: https://www.climatologylab.org/terraclimate.html. The ERA5 soil moisture data are from: https://cds.climate.copernicus.eu/datasets/reanalysis‐era5‐land‐monthly‐means?tab=overview. GIMMS LAI4g data are from: https://doi.org/10.5281/zenodo.7649108. The species diversity data are from: https://anthroecology.org/anthromes/plantbiodiversity/. The SLA data are from: https://gee‐community‐catalog.org/projects/ltrait/. The rooting depth data are from: https://doi.org/10.5281/zenodo.10885724. The forest canopy height data are from: https://csdms.colorado.edu/wiki/Alldata:Global_Forest_Heights. The wood density data are from: https://doi.org/10.5281/zenodo.10804643. All analyses are done with the open‐source software R 4.4.2. The code and data are available via Figshare at https://doi.org/10.6084/m9.figshare.28826543.
