# Supplementary material for: A Global Comparison of Direct and Legacy Effects of Drought on Ecosystem Productivity
Source: Ecol Lett. 2026 May 3;29:e70390. doi: 10.1111/ele.70390 (PMC13136783; doi:10.1111/ele.70390)
Supplement: Supplementary file 1 — Figure S1: A schematic of pre‐ and postdrought data. There are three predrought years and four postdrought years to derive the change in the magnitude of GPP due to the direct and legacy effects. Figure S2: Accuracy of random forest regression for the training and testing datasets. Comparison of observed GPP from flux tower data and predicted GPP from random forest regression, where bias is the observed GPP minus the predicted GPP. p value comes from linear regression. Figure S3: Comparison of the legacy effect‐induced change in GPP (ΔGPPlag) based on predrought GPP and random forest prediction. (a, b) ΔGPPlag based on predrought GPP versus ΔGPPlag based on (a) GPP anomaly and (b) GPP from random forest regression. GPP anomaly is calculated using annual GPP minus the average GPP for each site. The p values are based on linear regression. Figure S4: Comparison of 1, 2, 3, and 4‐year legacy effect‐induced relative change in GPP. (a, b) Direct and legacy effects induced relative change when using predrought GPP as the expected GPP and using (a) PDSI and (b) CWD to identify drought. Different durations, including 1, 2, 3, and 4‐year, are used for comparisons. (c, d) Relative change in GPP when using random forest regression to estimate the expected GPP and using (c) PDSI and (d) CWD to identify drought. The dots are the median changes, and the error bars are the corresponding 95% confidence intervals. Asterisks (*) indicate the confidence intervals do not cross zero. The numbers at the bottom are the sample size. Figure S5: Relative change in GPP in response to direct and legacy effects across land covers. (a–c) Relative change in GPP across aridity levels due to (a) the direct effect, (b) legacy effect based on predrought GPP, and (c) legacy effect based on random forest predicted GPP. (d–f) Relative change in GPP across land cover types due to (d) the direct effect, (e) legacy effect based on predrought GPP, and (f) legacy effect based on random forest predicted GPP. [file ELE-29-0-s001.pdf]

Supporting Information

A global comparison of direct and legacy effects of drought on ecosystem productivity

Meng Liu[1,2*], Steven A. Kannenberg[3], Josep Peñuelas[4,5], William R. L. Anderegg[1,2]

Author affiliations: [1]School of Biological Sciences, University of Utah, Salt Lake City, UT, USA; [2]Wilkes Center for Climate Science and Policy, University of Utah, Salt Lake City, UT, USA; [3]Department of Biology, West Virginia University, Morgantown, WV, USA; [4]CREAF, Cerdanyola del Vallès, Barcelona, Catalonia, Spain; [5]CSIC, Global Ecology Unit CREAF-CSIC-UAB, Bellaterra, Barcelona, Catalonia, Spain

This PDF file includes:

Figures S1 to S6
Tables S1 to S3

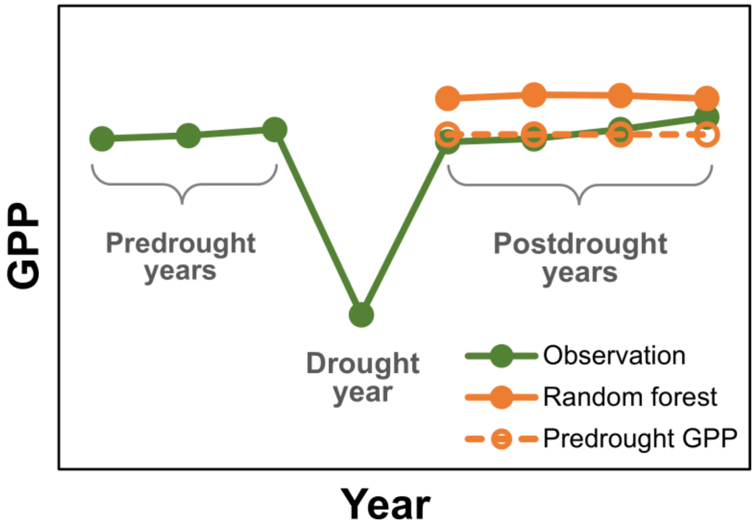

Figure S1 A schematic of pre- and postdrought data. There are three predrought years and four postdrought years to derive the change in the magnitude of GPP due to the direct and legacy effects.

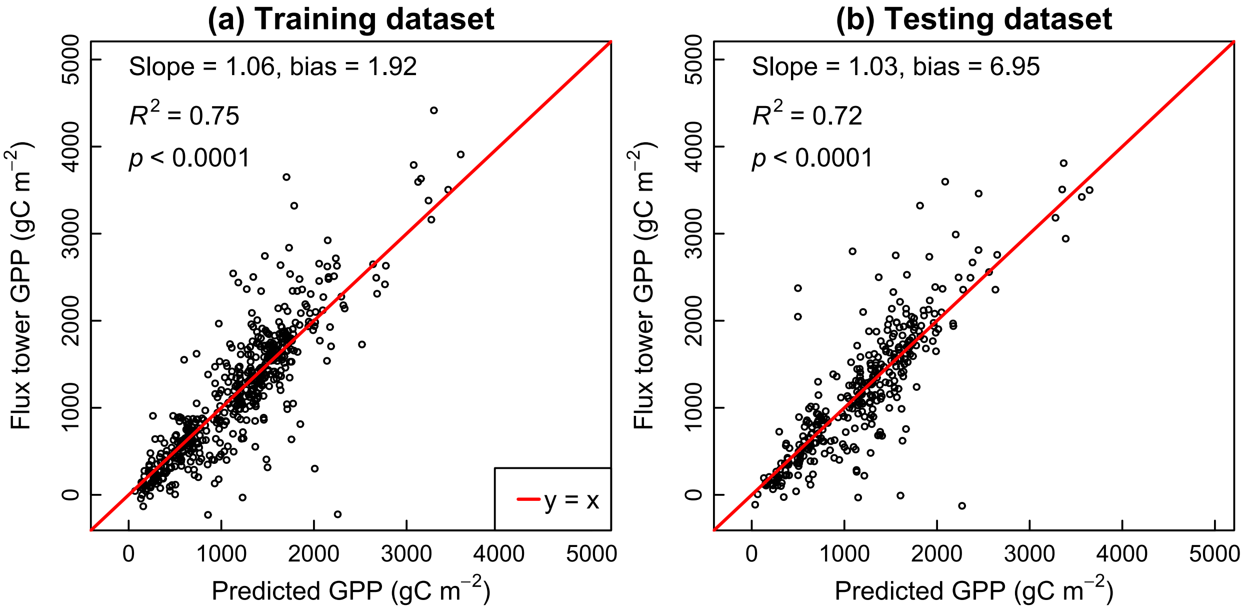

Figure S2 Accuracy of random forest regression for the training and testing datasets. Comparison of observed GPP from flux tower data and predicted GPP from random forest regression, where bias is the observed GPP minus the predicted GPP. *p* value comes from linear regression.

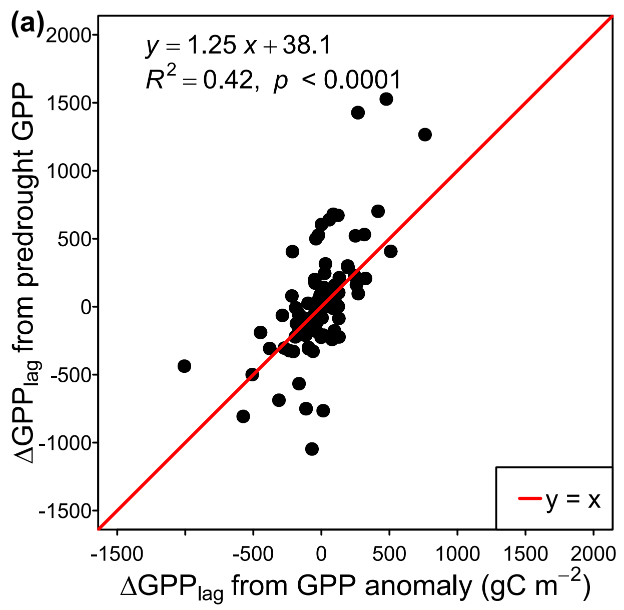

Figure S3 Comparison of the legacy effect-induced change in GPP (ΔGPP_{lag}) based on predrought GPP and random forest prediction. (**a-b**) ΔGPP_{lag} based on predrought GPP vs ΔGPP_{lag} based on (**a**) GPP anomaly and (**b**) GPP from random forest regression. GPP anomaly is calculated using annual GPP minus the average GPP for each site. The *p* values are based on linear regression.

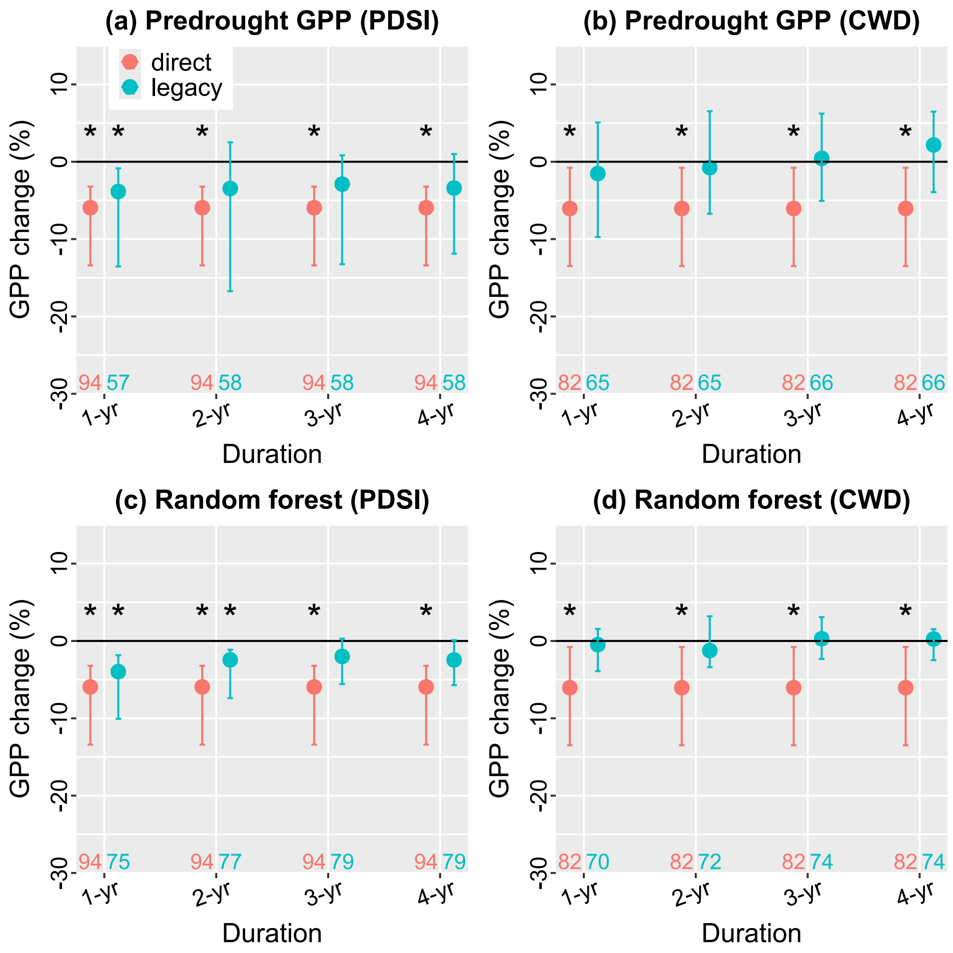

Figure S4 Comparison of 1-yr, 2-yr, 3-yr, and 4-yr legacy effect-induced relative change in GPP. (**a-b**) Direct and legacy effects induced relative change when using predrought GPP as the expected GPP and using (**a**) PDSI and (**b**) CWD to identify drought. Different durations, including 1-yr, 2-yr, 3-yr, and 4-yr, are used for comparisons. (**c-d**) Relative change in GPP when using random forest regression to estimate the expected GPP and using (**c**) PDSI and (**d**) CWD to identify drought. The dots are the median changes, and the error bars are the corresponding 95% confidence intervals. Asterisks (*) indicate the confidence intervals do not cross zero. The numbers at the bottom are the sample size.

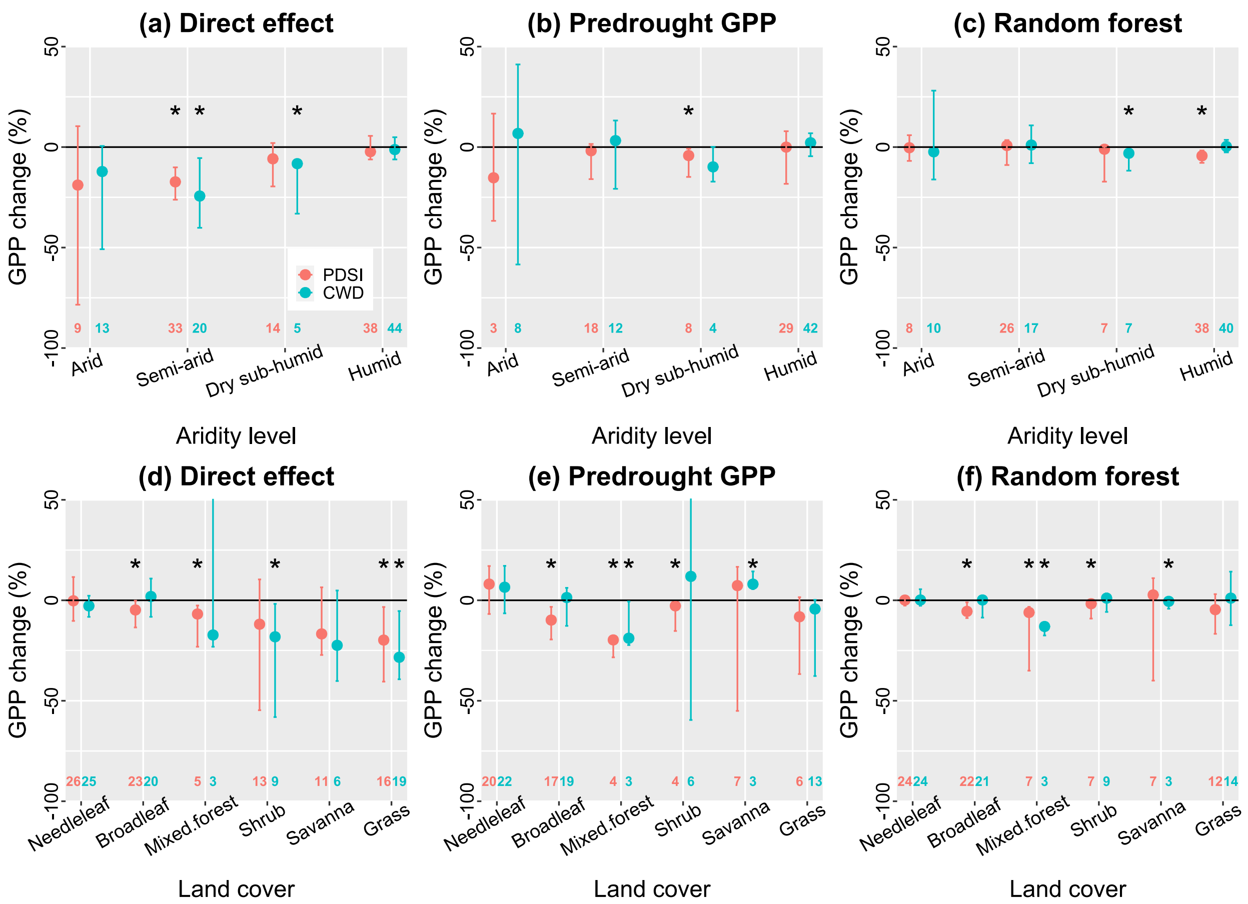

Figure S5 Relative change in GPP in response to direct and legacy effects across land covers. (**a-c**) Relative change in GPP across aridity levels due to (**a**) the direct effect, (**b**) legacy effect based on predrought GPP, and (**c**) legacy effect based on random forest predicted GPP. (**d-f**) Relative change in GPP across land cover types due to (**d**) the direct effect, (**e**) legacy effect based on predrought GPP, and (**f**) legacy effect based on random forest predicted GPP. Three years predrought and four years postdrought are used. The dots are the median changes, and the error bars are the corresponding 95% confidence intervals. Asterisks (*) indicate the confidence intervals do not cross zero. The numbers at the bottom are the sample size.

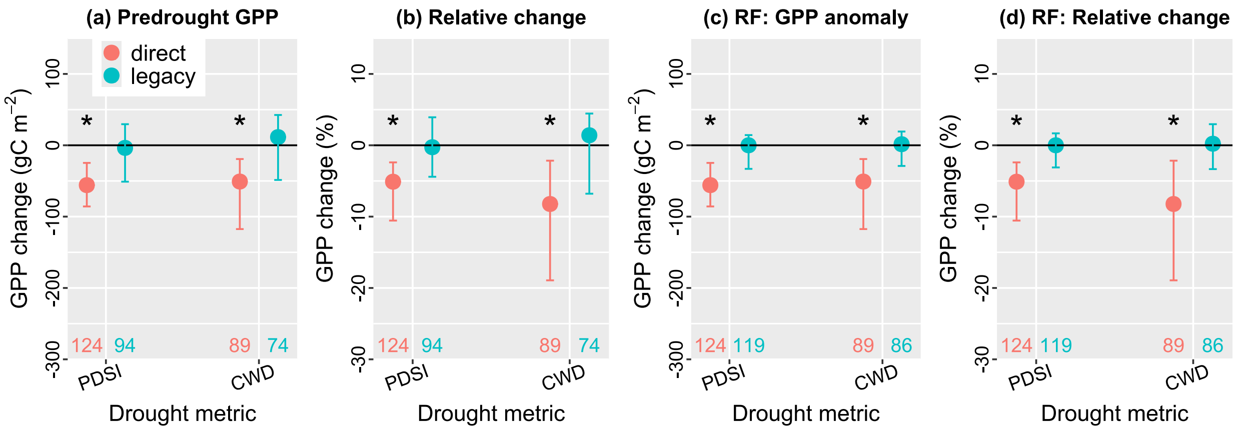

Figure S6 Direct and legacy effects on the magnitude of growing season GPP. (**a-b**) The direct effect-induced (ΔGPP_{dir}) and legacy effect-induced (ΔGPP_{lag}) (**a**) change in GPP and (**b**) relative change in GPP when using predrought GPP as the expected GPP. (**c-d**) Use random forest (RF) regression to estimate the expected GPP and further calculate ΔGPP_{dir} and ΔGPP_{lag} based on (**c**) GPP anomaly and (**d**) relative change. Four years postdrought are used. The growing season is from April to October in the Northern Hemisphere (> 23.5°N), October to April in the Southern Hemisphere (< 23.5°S), and the whole year in the tropics. GPP, PDSI, and CWD are all extracted from the growing season. Three years predrought and four years postdrought are used. The dots are the median changes, and the error bars are the corresponding 95% confidence intervals. Asterisks (*) indicate the confidence intervals do not cross zero. The numbers at the bottom are the sample size.

Table S1. Flux tower sites from FLUXNET2015 and AmeriFlux FLUXNET.

Site ID	LOCATION_LAT	LOCATION_LONG	IGBP
AR-SLu	-33.4648	-66.4598	MF
AR-Vir	-28.2395	-56.1886	ENF
AT-Neu	47.1167	11.3175	GRA
AU-Ade	-13.0769	131.1178	WSA
AU-ASM	-22.283	133.249	SAV
AU-Cpr	-34.0021	140.5891	SAV
AU-Cum	-33.6152	150.7236	EBF
AU-DaP	-14.0633	131.3181	GRA
AU-DaS	-14.1593	131.3881	SAV
AU-Dry	-15.2588	132.3706	SAV
AU-Emr	-23.8587	148.4746	GRA
AU-Gin	-31.3764	115.7138	WSA
AU-GWW	-30.1913	120.6541	SAV
AU-How	-12.4943	131.1523	WSA
AU-Lox	-34.4704	140.6551	DBF
AU-RDF	-14.5636	132.4776	WSA
AU-Rig	-36.6499	145.5759	GRA
AU-Rob	-17.1175	145.6301	EBF
AU-Stp	-17.1507	133.3502	GRA
AU-TTE	-22.287	133.64	GRA
AU-Tum	-35.6566	148.1517	EBF
AU-Wac	-37.4259	145.1878	EBF
AU-Whr	-36.6732	145.0294	EBF
AU-Wom	-37.4222	144.0944	EBF
AU-Ync	-34.9893	146.2907	GRA
BE-Bra	51.3076	4.5198	MF
BE-Vie	50.3049	5.9981	MF
BR-Sa1	-2.8567	-54.9589	EBF
BR-Sa3	-3.018	-54.9714	EBF
CA-Gro	48.2167	-82.1556	MF
CA-Man	55.8796	-98.4808	ENF
CA-NS1	55.8792	-98.4839	ENF
CA-NS2	55.9058	-98.5247	ENF
CA-NS3	55.9117	-98.3822	ENF
CA-NS4	55.9144	-98.3806	ENF
CA-NS5	55.8631	-98.485	ENF
CA-NS6	55.9167	-98.9644	OSH
CA-NS7	56.6358	-99.9483	OSH
CA-Oas	53.6289	-106.198	DBF
CA-Obs	53.9872	-105.118	ENF
CA-Qfo	49.6925	-74.3421	ENF

CA-SF1	54.485	-105.818	ENF
CA-SF2	54.2539	-105.878	ENF
CA-SF3	54.0916	-106.005	OSH
CA-TP1	42.6609	-80.5595	ENF
CA-TP2	42.7744	-80.4588	ENF
CA-TP3	42.7068	-80.3483	ENF
CA-TP4	42.7102	-80.3574	ENF
CA-TPD	42.6353	-80.5577	DBF
CG-Tch	-4.2892	11.6564	SAV
CH-Cha	47.2102	8.4104	GRA
CH-Dav	46.8153	9.8559	ENF
CH-Fru	47.1158	8.5378	GRA
CH-Lae	47.4783	8.3644	MF
CH-Oe1	47.2858	7.7319	GRA
CN-Cha	42.4025	128.0958	MF
CN-Cng	44.5934	123.5092	GRA
CN-Dan	30.4978	91.0664	GRA
CN-Din	23.1733	112.5361	EBF
CN-Du2	42.0467	116.2836	GRA
CN-Du3	42.0551	116.2809	GRA
CN-HaM	37.37	101.18	GRA
CN-Qia	26.7414	115.0581	ENF
CN-Sw2	41.7902	111.8971	GRA
CZ-BK1	49.5021	18.5369	ENF
CZ-BK2	49.4944	18.5429	GRA
DE-Gri	50.95	13.5126	GRA
DE-Hai	51.0792	10.4522	DBF
DE-Lkb	49.0996	13.3047	ENF
DE-Lnf	51.3282	10.3678	DBF
DE-Obe	50.7867	13.7213	ENF
DE-RuR	50.6219	6.3041	GRA
DE-Tha	50.9626	13.5651	ENF
DK-Eng	55.6905	12.1918	GRA
DK-Sor	55.4859	11.6446	DBF
ES-Amo	36.8336	-2.2523	OSH
ES-LgS	37.0979	-2.9658	OSH
ES-LJu	36.9266	-2.7521	OSH
ES-Ln2	36.9695	-3.4758	OSH
FI-Hyy	61.8474	24.2948	ENF
FI-Let	60.6418	23.9595	ENF
FI-Sod	67.3624	26.6386	ENF
FR-Fon	48.4764	2.7801	DBF
FR-LBr	44.7171	-0.7693	ENF
FR-Pue	43.7413	3.5957	EBF

GF-Guy	5.2788	-52.9249	EBF
GH-Ank	5.2685	-2.6942	EBF
GL-ZaH	74.4733	-20.5503	GRA
IT-CA1	42.3804	12.0266	DBF
IT-CA3	42.38	12.0222	DBF
IT-Col	41.8494	13.5881	DBF
IT-Cp2	41.7043	12.3573	EBF
IT-Cpz	41.7052	12.3761	EBF
IT-Isp	45.8126	8.6336	DBF
IT-La2	45.9542	11.2853	ENF
IT-Lav	45.9562	11.2813	ENF
IT-MBo	46.0147	11.0458	GRA
IT-Noe	40.6062	8.1517	CSH
IT-PT1	45.2009	9.061	DBF
IT-Ren	46.5869	11.4337	ENF
IT-Ro1	42.4081	11.93	DBF
IT-Ro2	42.3903	11.9209	DBF
IT-SR2	43.732	10.2909	ENF
IT-SRo	43.7279	10.2844	ENF
IT-Tor	45.8444	7.5781	GRA
JP-MBF	44.3869	142.3186	DBF
JP-SMF	35.2617	137.0788	MF
MY-PSO	2.973	102.3062	EBF
NL-Hor	52.2403	5.0713	GRA
NL-Loo	52.1666	5.7436	ENF
PA-SPn	9.3181	-79.6346	DBF
PA-SPs	9.3138	-79.6314	GRA
RU-Cok	70.8291	147.4943	OSH
RU-Fyo	56.4615	32.9221	ENF
RU-Ha1	54.7252	90.0022	GRA
SD-Dem	13.2829	30.4783	SAV
SN-Dhr	15.4028	-15.4322	SAV
US-ARc	35.5465	-98.04	GRA
US-Blo	38.8953	-120.633	ENF
US-Cop	38.09	-109.39	GRA
US-GBT	41.3658	-106.24	ENF
US-GLE	41.3665	-106.24	ENF
US-Goo	34.2547	-89.8735	GRA
US-Ha1	42.5378	-72.1715	DBF
US-IB2	41.8406	-88.241	GRA
US-KS1	28.4583	-80.6709	ENF
US-KS2	28.6086	-80.6715	CSH
US-LWW	34.9604	-97.9789	GRA
US-Me1	44.5794	-121.5	ENF

US-Me2	44.4523	-121.557	ENF
US-Me3	44.3154	-121.608	ENF
US-Me4	44.4992	-121.622	ENF
US-Me5	44.4372	-121.567	ENF
US-Me6	44.3233	-121.608	ENF
US-MMS	39.3232	-86.4131	DBF
US-NR1	40.0329	-105.546	ENF
US-Oho	41.5545	-83.8438	DBF
US-PFa	45.9459	-90.2723	MF
US-Prr	65.1237	-147.488	ENF
US-SRC	31.9083	-110.84	OSH
US-SRG	31.7894	-110.828	GRA
US-SRM	31.8214	-110.866	WSA
US-Sta	41.3966	-106.802	OSH
US-Syv	46.242	-89.3477	MF
US-Ton	38.4309	-120.966	WSA
US-UMB	45.5598	-84.7138	DBF
US-UMd	45.5625	-84.6975	DBF
US-Var	38.4133	-120.951	GRA
US-WCr	45.8059	-90.0799	DBF
US-Whs	31.7438	-110.052	OSH
US-Wi0	46.6188	-91.0814	ENF
US-Wi1	46.7305	-91.2329	DBF
US-Wi2	46.6869	-91.1528	ENF
US-Wi3	46.6347	-91.0987	DBF
US-Wi4	46.7393	-91.1663	ENF
US-Wi5	46.6531	-91.0858	ENF
US-Wi6	46.6249	-91.2982	OSH
US-Wi7	46.6491	-91.0693	OSH
US-Wi8	46.7223	-91.2524	DBF
US-Wi9	46.7385	-91.0746	ENF
US-Wkg	31.7365	-109.942	GRA
ZM-Mon	-15.4391	23.2525	DBF
BR-CST	-7.9682	-38.3842	DNF
BR-Npw	-16.498	-56.412	WSA
CA-Ca1	49.8673	-125.334	ENF
CA-Ca2	49.8705	-125.291	ENF
CA-Cbo	44.3167	-79.9333	DBF
CA-LP1	55.1119	-122.841	ENF
CA-MA3	50.1774	-97.8686	GRA
MX-Tes	27.8446	-109.298	DBF
US-A32	36.8193	-97.8198	GRA
US-Bar	44.0646	-71.2881	DBF
US-BRG	39.2167	-86.5406	GRA

US-BZS	64.6963	-148.324	ENF
US-CS2	44.1467	-89.5002	ENF
US-EML	63.8784	-149.254	OSH
US-Fmf	35.1426	-111.727	ENF
US-Fuf	35.089	-111.762	ENF
US-HB2	33.3242	-79.244	ENF
US-HB3	33.3482	-79.2322	ENF
US-Hn2	46.6889	-119.464	GRA
US-Hn3	46.6878	-119.461	OSH
US-Ho2	45.2091	-68.747	ENF
US-ICh	68.6068	-149.296	OSH
US-ICt	68.6063	-149.304	OSH
US-Jo1	32.582	-106.635	OSH
US-Jo2	32.5849	-106.603	OSH
US-KFS	39.0561	-95.1907	GRA
US-KLS	38.7745	-97.5684	GRA
US-Kon	39.0824	-96.5603	GRA
US-LS2	31.5659	-110.134	SAV
US-Mo2	38.9488	-91.9945	GRA
US-MOz	38.7441	-92.2	DBF
US-Mpj	34.4385	-106.238	WSA
US-NC1	35.8118	-76.7119	ENF
US-NC3	35.799	-76.656	ENF
US-NGC	64.8618	-163.7	GRA
US-ONA	27.3836	-81.9509	GRA
US-Rls	43.1439	-116.736	CSH
US-Rms	43.0645	-116.749	CSH
US-Ro4	44.6781	-93.0723	GRA
US-Rpf	65.1198	-147.429	DBF
US-Rwe	43.0653	-116.759	CSH
US-Rwf	43.1207	-116.723	CSH
US-Rws	43.1675	-116.713	OSH
US-Seg	34.3623	-106.702	GRA
US-Ses	34.3349	-106.744	OSH
US-Sne	38.0369	-121.755	GRA
US-Snf	38.0402	-121.727	GRA
US-SRS	31.8173	-110.851	WSA
US-Vcm	35.8884	-106.532	ENF
US-Vcp	35.8642	-106.597	ENF
US-Wjs	34.4255	-105.862	SAV
US-xAB	45.7624	-122.33	ENF
US-xAE	35.4106	-99.0588	GRA
US-xBL	39.0603	-78.0716	DBF
US-xBN	65.154	-147.503	ENF

US-xBR	44.0639	-71.2873	DBF
US-xCL	33.4012	-97.57	GRA
US-xCP	40.8155	-104.746	GRA
US-xDC	47.1617	-99.1066	GRA
US-xDJ	63.8811	-145.751	ENF
US-xDL	32.5417	-87.8039	MF
US-xGR	35.689	-83.5019	DBF
US-xHA	42.5369	-72.1727	DBF
US-xHE	63.8757	-149.213	OSH
US-xJE	31.1948	-84.4686	ENF
US-xJR	32.5907	-106.843	OSH
US-xKA	39.1104	-96.6129	GRA
US-xKZ	39.1008	-96.5631	GRA
US-xMB	38.2483	-109.388	OSH
US-xML	37.3783	-80.5248	DBF
US-xNG	46.7697	-100.915	GRA
US-xNQ	40.1776	-112.452	OSH
US-xRM	40.2759	-105.546	ENF
US-xSB	29.6893	-81.9934	ENF
US-xSC	38.8929	-78.1395	DBF
US-xSE	38.8901	-76.56	DBF
US-xSJ	37.1088	-119.732	SAV
US-xSR	31.9107	-110.836	OSH
US-xST	45.5089	-89.5864	DBF
US-xTA	32.9505	-87.3933	ENF
US-xTR	45.4937	-89.5857	DBF
US-xUK	39.0404	-95.1921	DBF
US-xUN	46.2339	-89.5373	MF
US-xWD	47.1282	-99.2414	GRA
US-xYE	44.9535	-110.539	ENF

Table S2. A summary of variables used in the manuscript.

Abbreviations	Definitions	Data sources
ΔGPP_{dir}	Direct effect-induced change in GPP	Flux tower GPP
ΔGPP_{lag}	Legacy effect-induced change in GPP	Flux tower GPP
$GPP_{drought}$	Mean annual GPP during drought years	Flux tower GPP
$GPP_{predrought}$	Mean annual GPP in predrought years	Flux tower GPP
$GPP_{postdrought}$	Mean annual GPP in postdrought years	Flux tower GPP
$GPP_{expected}$	GPP we expected assuming the drought didn't occur	Flux tower GPP or modeling
CWD	Climatic water deficit	TerraClimate
PDSI	Palmer Drought Severity Index	TerraClimate
Srad	Downward surface shortwave radiation	TerraClimate
VPD	Vapor pressure deficit	TerraClimate
T	Temperature	TerraClimate
P	Precipitation	TerraClimate
SM	Soil moisture	ERA5
LAI	Leaf area index	Cao et al. 2023
SOC	Soil organic carbon	SoilGrids250m
Soil.N	Soil nitrogen	SoilGrids250m
Soil.CEC	Soil cation exchange capacity	SoilGrids250m
AI	Aridity index	Zomer et al. 2022
$PDSI_{postdrought}$	Mean annual PDSI in postdrought years	TerraClimate
$PDSI_{drought}$	Mean annual PDSI during drought years	TerraClimate
$\Delta PDSI_{post-pre}$	$PDSI_{postdrought}$ minus predrought mean annual PDSI	TerraClimate
$\Delta PDSI_{drought-pre}$	$PDSI_{drought}$ minus predrought mean annual PDSI	TerraClimate
$Srad_{postdrought}$	Mean annual Srad in postdrought years	TerraClimate
$Srad_{drought}$	Mean annual Srad during drought years	TerraClimate
$\Delta Srad_{post-pre}$	$Srad_{postdrought}$ minus predrought mean annual Srad	TerraClimate
$\Delta Srad_{drought-pre}$	$Srad_{drought}$ minus predrought mean annual Srad	TerraClimate
$VPD_{postdrought}$	Mean annual VPD in postdrought years	TerraClimate
$VPD_{drought}$	Mean annual VPD during drought years	TerraClimate
$\Delta VPD_{post-pre}$	$VPD_{postdrought}$ minus predrought mean annual VPD	TerraClimate
$\Delta VPD_{drought-pre}$	$VPD_{drought}$ minus predrought mean annual VPD	TerraClimate
$T_{postdrought}$	Mean annual temperature (T) in postdrought years	TerraClimate
$T_{drought}$	Mean annual T during drought years	TerraClimate
$\Delta T_{post-pre}$	$T_{postdrought}$ minus predrought mean annual T	TerraClimate
$\Delta T_{drought-pre}$	$T_{drought}$ minus predrought mean annual T	TerraClimate
$P_{postdrought}$	Mean annual precipitation (P) in postdrought years	TerraClimate
$P_{drought}$	Mean annual P during drought years	TerraClimate
$\Delta P_{post-pre}$	$P_{postdrought}$ minus predrought mean annual P	TerraClimate
$\Delta P_{drought-pre}$	$P_{drought}$ minus predrought mean annual P	TerraClimate
$SM_{postdrought}$	Mean annual soil moisture (SM) in postdrought years	ERA5
$SM_{drought}$	Mean annual SM during drought years	ERA5
$\Delta SM_{post-pre}$	$SM_{postdrought}$ minus predrought mean annual SM	ERA5
$\Delta SM_{drought-pre}$	$SM_{drought}$ minus predrought mean annual SM	ERA5
$LAI_{postdrought}$	Mean annual LAI in postdrought years	Cao et al. 2023
$LAI_{drought}$	Mean annual LAI during drought years	Cao et al. 2023
$\Delta LAI_{post-pre}$	$LAI_{postdrought}$ minus predrought mean annual LAI	Cao et al. 2023
$\Delta LAI_{drought-pre}$	$LAI_{drought}$ minus predrought mean annual LAI	Cao et al. 2023
diversity	Species diversity	Ellis et al. 2012
SLA	Specific leaf area	Moreno-Martínez et al. 2018
root.depth	Rooting depth	Stocker et al. 2023
root.shoot	Root shoot ratio	Ma et al. 2021
wood.density	Wood density	Yang et al. 2024
canopy.height	Forest canopy height	Simard et al. 2011
land.cover	IGBP land cover	Flux tower site info

Table S3. Results of the generalized additive models (GAM) for the direct and indirect effect-induced GPP change.

	**Response variable**	**Smooth terms**	**edf**	**F**	***p*-value**
**For direct effect**	ΔGPP_{dir}	land.cover			0.0003
R^2 = 0.38		s($T_{drought}$)	1.48	2.58	0.024
		s(root.depth)	1.69	3.18	0.017
		s($\Delta LAI_{drought\text{-}pre}$)	1.06	3.06	0.0086
		s(SLA)	1.17	4.92	0.001
		s(diversity)	0.85	2.54	0.004
		s(canopy.height)	0.67	1.17	0.039
**For indirect effect**	ΔGPP_{ind}	s(ΔGPP_{dir})	1.71	19.38	0.0
R^2 = 0.49		s($\Delta T_{post\text{-}pre}$)	0.89	2.61	0.0097
		s(root.depth)	1.12	2.21	0.02
		s($\Delta VPD_{post\text{-}pre}$)	1.11	3.48	0.004
		s(Soil.N)	1.04	2.65	0.011
		s($\Delta PDSI_{post\text{-}pre}$)	0.99	2.88	0.0091
